# Academic influencers: Clinical and Translational Science scholars and trainees at the intersection of influential scholarship and public attention

**DOI:** 10.1017/cts.2025.10067

**Published:** 2025-06-02

**Authors:** Eric J. Nehl, Clara M. Pelfrey, Deborah DiazGranados, Gaurav Dave, Nicole M. Llewellyn

**Affiliations:** 1 Georgia Clinical and Translational Science Alliance, Emory University School of Medicine, Atlanta, GA, USA; 2 Emory University Rollins School of Public Health, Atlanta, GA, USA; 3 Case Western Reserve University, Cleveland, OH, USA; 4 Virginia Commonwealth University, Psychiatry, Wright Regional Center for Clinical and Translational Science, Richmond, VA, USA; 5 University of North Carolina, Chapel Hill, NC, USA

**Keywords:** Bibliometrics, altmetrics, research impact, research education, translational science

## Abstract

**Introduction:**

Clinical and Translational Science trainees are motivated to publish influential research. However, the extent to which this work gains influence with the public is largely unknown.

**Methods:**

The authors identified over 30,000 publications that received KL2 or TL1 grant support through a Clinical and Translational Science Awards hub, from 2006 through January 2024. The Altmetric Explorer database was then used, to collect references in sources such as news articles, tweets, and blogs. We investigated bibliometric characteristics and content areas, provide illustrative examples of influence, and determine the characteristics most likely to gain public attention.

**Results:**

Articles were published in 3,923 journals with a mean Journal Impact Factor (JIF) of 5.78, a mean Relative Citation Ratio (RCR) score of 2.02, and were cited an average of 33.7 times, totaling 1,017,291 citations. Over 4,800 were referenced in policy and were mentioned in over 64K news articles, 7K blog posts, and 480K X (Twitter) posts. The mean Altmetric Attention Score was 28.9, with 18.5% having scores of 20 or higher. Nearly 30% were related to COVID-19, indicating close public attention to this important health topic. Regression analyses indicate that higher JIF, being published after 2020, receiving more Mendeley downloads, higher RCR scores, being cited by in policy, and fewer academic citations, were more likely to receive altmetric attention.

**Conclusions:**

By demonstrating how supported research has influence beyond academia to become “Academic Influencers,” this study represents a significant advance in our ability to evaluate translational research impact.

## Introduction

Some peer-reviewed research articles are disseminated widely in society, gaining traction and popularity with the public through various forms of news and social media. Others may never be recognized beyond their academic fields, while still having the potential to accumulate influence and impact within those fields over time. A third unique subset of research articles pairs public attention and academic impact, acting as “Academic Influencers” through their popularity among both the research community and larger society. Articles may gain this impact in many ways, including because they contribute a substantial scientific advancement, solve a public health need, are from a notable scientist(s) or journal, represent a topic of widespread societal interest, or because they support political viewpoints or agendas [1–6]. Or, as in the case of scientific research related to COVID-19, there may be an overwhelming combination of all the aforementioned factors [1,7,8]. This study examined altmetrics- alternative publication metrics which, rather than measuring scholarly influence through academic citations, measure public attention through references in the news media, online discourse and social media, policy and legislative literature, technological patents, and clinical guidelines. This study’s main goal is to use altmetric tools to understand why a research article or group of articles have influence beyond academia, in the broader public sphere. Bibliometrics offer a straightforward way to support the evaluation of Clinical and Translational Science (CTS) through a flexible set of methodological tools and measures that allow for a comprehensive examination of research publications. Using bibliometrics, CTS researchers have been able to measure the broader impacts of research on clinical and community practice, return on investments in science, public legislation, and policy [9]. These adaptable bibliometric designs have investigated both examinations of whole fields of research and focused case studies of the successes and failures of the research enterprise [1,2,4,9–11]. One growing area of bibliometric research that can be used to understand the influence of research beyond academia, such as news media and community influence public reach of research is altmetrics [10,11].

The research conducted and disseminated from researchers within academic medical centers through peer-reviewed journals is a fundamental building block to advancing healthcare and community public health practice. Producing this science is complex, difficult, requires significant vision, and outcomes often require long-term interdisciplinary efforts [12,13]. The Clinical and Translational Science Awards (CTSA) consortium, with funding from the National Institutes of Health (NIH) through the National Center for Advancing Translational Sciences (NCATS), aims to accelerate the translational process that moves observations and discoveries from laboratory benches to patients in clinics and their communities; including disseminating research in alternative ways that build the support and confidence of the diverse audiences beyond academia [14–17]. NCATS supports innovative medical research via a consortium of more than 60 translational research program hubs (i.e. CTSA hubs) across the nation [18]. These CTSA hubs organize institutional research resources, accelerate CTS production, and are at the forefront of training the next generation of translational scientists.

CTS training programs based within these academic institutions must be innovative, evidence-based, comprehensive, and responsive to the emerging needs of CTS scholars and trainees [12,19]. Foundational training and promotion of trainees’ research through dissemination in scientific journals is critical to the success of CTS, as the scholars and trainees will serve as the future leaders in research and community engagement to improve health outcomes. NCATS, through their network of funded CTSA hubs, provides a range of research training and mentored career awards for predoctoral students, postdoctoral fellows, and early-stage investigators, including Masters degrees, certificates programs, T Awards (TL1 or T32 pre and post doc), and KL2 Awards, which provide foundational skills and mentoring to promote expertise and capacity in CTS [12,20]. Significant national initiatives and evaluation efforts have assessed the outcomes of CTS training on scholar and trainee careers, with indications that career trajectories are greatly enhanced through these training programs [21–29]. For example, previous research has shown that those who have received NCATS KL2 funding obtained subsequent independent research (R01) award faster than an equivalent group of early career faculty [30]. However, a large-scale evaluation of the publication output of CTS scholar and trainee publications has not been conducted. CTS trainees are motivated to publish impactful research articles to help build their reputation and credibility within their fields, secure funding, be competitive for faculty appointments, and eventually gain promotions and tenure [31,32]. Although becoming an academic influencer or publishing influencer articles may not be a personal priority for all scholars and trainees, they are often expected by their mentors, institutions, and granting agencies to disseminate articles in high-impact journals that demonstrate quality and quantity as measured by traditional impact measures and newer metrics of science dissemination [31–33].

A key goal of science dissemination is to communicate science advances beyond academia to the public. A variety of frameworks have been presented to help researchers engage those outside of academia and to “Develop, Demonstrate, and Disseminate” innovations [16,34–36]. Bibliometric methods allow for a structured evaluation into trainee researchers that emerge as academic influencers. We conducted our evaluation using three complementary approaches, aiming to: (1) evaluate bibliometric characteristics and content of the CTSA training grant-supported publication portfolio that has amassed since the inception of the CTSA program in 2006, including altmetrics that reflect public attention and interest/engagement; (2) provide illustrative case examples of CTSA training grant-supported research that generated high levels of interest and impact outside academic spheres (academic influencers); and (3) determine the characteristics of articles that are most likely to gain public altmetric attention.

## Materials and methods

### Data collection

This study includes publications authored by scholars and trainees who acknowledged CTSA KL2 or TL1 grant support for their research from any of the 66 CTSA hubs operating across 33 states in the United States. Investigators are asked to cite their respective institutions’ CTSA grants in publications that result from support received during their research. Although this likely results in an undercount of all supported research, it is a verifiable and reproducible measure of research supported by significant CTSA resources and is consistent with criteria for reporting supported products to the NIH. Data were collected in January 2024. We compiled CTSA hub grant project numbers from NIH RePORTER [37], including past and present KL2, TL1^a^, and supplemental awards funded by NCATS and its predecessor, the National Center for Research Resources. Although in 2023 NCATS transitioned to K12 and T32 award mechanisms in the latest Funding Opportunity Announcement, no publications had acknowledged these support mechanisms at the time of data collection for this study. Using PubMed [38], we identified 30,217 publications that cited a CTSA KL2 or TL1 grant since they were established in 2006 through January 2024.

This study was interested primarily in bibliometrics at the intersection of academic and public attention, policy, research areas and topics. To retrieve journal and content information, the list of NCATS-supported publications was first searched in Clarivate Analytics Web of Science’s (WoS) subscription-based InCites application [39]. To retrieve year, citation and translational feature information, the list of publications was searched in the NIH’s iCite application [40]. To retrieve author and altmetric information, the list of publications was searched in Digital Science’s subscription-based Dimensions application [41]. Finally, publications were queried in Overton, which, at the time of writing, encompasses a growing database of over 13 million policy documents from over 1,000 nonacademic organizations [42].

## Measures

### InCites

**Journal Impact Factor.** Journal Impact Factor (JIF) data were available from InCites and collected for 25,588 articles (84.7%); very small or recently established journals may not be indexed yet by InCites. JIF is an unadjusted measure of typical citation rates for the journals in which articles were published over the previous 2 years, (e.g., a JIF of 5 means that articles published in that journal in the past 2 years were cited an average of 5 times) [43].

**WoS Research Areas.** The InCites application includes multiple schemes for classifying publications according to research content area. For each publication in our data set, we examined: the WoS research area (WoSRA) scheme, which was available for all 28,474 articles indexed in InCites (94.2%); the most granular categorization scheme for research content area available from InCites, which includes 252 subject categories across science, social science, arts and humanities; not all of which are expected to be applicable to clinical/translational pediatric research. The WoSRA is usually assigned based upon the content area of the journal in which the article is published. If the journal is general or multidisciplinary (e.g., New England Journal of Medicine, PlosOne, etc) then the article is assigned based upon its cited reference list and only assigned to the general category if no more specific designation can be made. It is typically not feasible to assign a journal/publication to a single category, therefore, up to six research areas may be assigned to a given journal and corresponding articles [39].

### iCite

**Publication Year.** Year of publication was collected to accommodate longitudinal analysis of research productivity and impact. Publication year was available for 100% of articles. Publication year was recoded into 2 categories for the third aim of this study, split into categories of pre-2020 and 2020 or after to explore the impact of COVID-19 on bibliometric indicators.

**Times Cited.** Total academic citation count was included as a measure of academic impact. Citation count was available for 100% of articles.

**Relative Citation Ratio**. Relative Citation Ratio (RCR) is a field-normalized citation metric that calculates the citation impact of an article relative to the average NIH-funded paper in its co-citation network [44]. The RCR indicates how many more citations a publication receives compared with others in their field (e.g., an RCR of **2.0** indicates that a publication is cited **2.0** times more than comparable publications). RCR data are available for publications that are at least 1 year old and was available for 28,332 articles (94%).

**Translational Features.** Article features related to translation include the a) Approximate Potential to Translate (APT) score [44], which uses a machine-learning approach to predict the percent likelihood that an article will eventually receive a clinical citation, assigning a value between 0.05 (no detectable signatures of translation) to 0.95 (extremely strong signatures of translation), b) the percentages of research involving human, animal, and molecular/cellular research as designated through the triangle of biomedicine [45] and c) designations as clinical articles and actual citations by clinical articles to date. Translational features were available for 100% of articles. Due to a nonnormal distribution, the APT was recoded into 2 categories based on a median split for the third aim of this study, with the median value placed in the lower category to achieve the most even split: high (> 50%) versus low (≤ 50%).

### Dimensions

**Altmetrics.** The Altmetric Attention Score (AAS) is a rank-ordered index score aggregated from several subcomponents that reflect media and community attention paid to an article and use of the article in public documents [46]. Subcomponents of the AAS detailed in this study include references in news articles, blog posts, policy, patent, F1000, Wikipedia, and X (formerly Twitter) posts. The AAS also includes references in Facebook, patent applications, policy documents (overlapping but not equivalent to those found in Overton) [47], and Wikipedia. AAS data are calculated for publications that are indexed by Altmetric Explorer and was available for 25,038 articles (82.9%). Additionally, the number of Mendeley Reference Management Program [48] reader downloads, an independent Altmetric measure that is not used in calculating the AAS was collected.

### Overton

**Policy.** We queried publications in Overton, which encompasses a growing database of millions of policy documents from nonacademic organizations (for policy document inclusion criteria, see help.overton.io) [42]. A total of 4,809 publications (15.9% of the overall portfolio) were found to be referenced in policy literature indexed by Overton. Use in policy was recoded into 2 categories for the third aim of this study: 1) used in policy, versus 2) not used in policy.

### Analytic plan

First, to summarize and provide context for the publication portfolio supported by the CTSA program, we conducted descriptive analyses compared by grant mechanism, with short- and long-term impact bibliometrics, including a longitudinal assessment of the total numbers of publications supported by the consortium, journal impact factors, APT scores, journals, and academic citations, as well as mean RCR. Additional metrics included policy literature citations and the numbers and percentages of articles represented by each research area. Lastly, we present short- and long-term altmetric impact measures for CTSA KL2- and TL1-supported publications, including AAS, and references in news stories, blog posts, X (posts, patent applications, F1000 peer-reviews, and Wikipedia pages.

Second, we identified 63 CTSA-supported publications with AAS scores greater than 1,000. We then selected case example articles from this group of articles representing a cross-section of time periods (e.g. pre-2020 and COVID-19 pandemic versus post-2020), research types, disease-foci, and modes of CTSA support. Using the full text of these selected articles, we provide illustrative examples from these top AAS articles by their research category, the CTSA hub which supported the research, the grant mechanism of support, and summarized the content and influence of this group of highly impactful publications.

Third, we assessed differences between CTSA-supported articles that received higher levels of altmetric attention, versus those that received less or no attention. Due to a non-normal distribution, the AAS was recoded into 3 categories based upon each article having received an AAS score of: zero (no attention), 1–20 (moderate attention), or greater than 20 (significant attention) [49]. We calculated descriptive statistics, Chi-square, and one-way ANOVAs to explore differences in bibliometric impact indicators. Key variables that were statistically significant in preliminary analyses were included in subsequent Polytomous Logit Universal Model (PLUM) regression analyses predicting the likelihood of receiving increasing altmetric attention. PLUM regressions account for the ordinal nature of altmetric attention and provide standard odds ratio estimates and significance tests. The data were analyzed using IBM SPSS Statistics for Windows, version 29.0 (IBM Corp., Armonk, N.Y., USA).

## Results

### Part 1: Characteristics and content of the CTSA training grant supported publication portfolio

**Figure 1.**
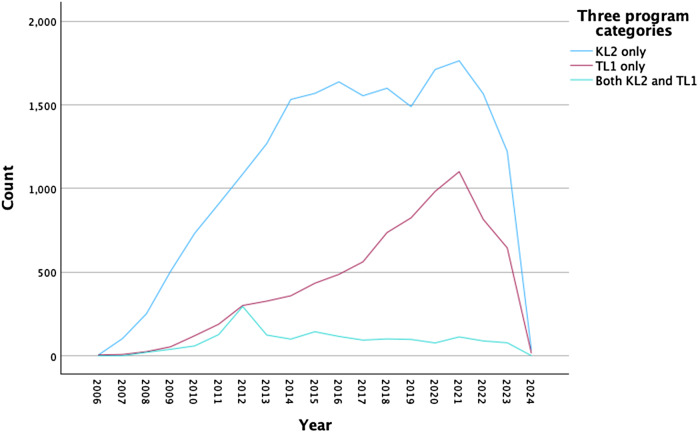
CTSA-supported KL2 and TL1 publication productivity over time by type of grant.

Of the 30,217 publications that met inclusion criteria, a majority (68%) cite only CTSA hub KL2 grants, 7,995 (26.5%) cite only TL1 training grants, and 1,676 (5.5%) cite both KL2 and TL1 grants. Figure 1 depicts the numbers of articles published across year intervals, showing a relatively consistent rise in publication productivity from 2006 through 2021, with a drop in productivity between 2021 and 2023 for all CTSA grant types.

The articles were published in 3,923 different journals with a mean article-level JIF of 5.78 (SD = 8.20, interquartile range = 2.65–6.07). The most frequent outlets included *PLoS One* (590 articles), *Scientific Reports* (198), *Journal of the American Geriatrics Society* (193), *Journal of General Internal Medicine* (186) *Clinical Infectious Diseases* (171), and *Cancer* (149). The articles were classified into 182 different Research Areas, the most frequent of which were *Oncology (2,362), Public, Environmental & Occupational health (2,190), Clinical Neurology (2056), Neurosciences (1976), and Surgery (1,874)*.

A key article-level bibliometric indicator is the RCR. The overall mean RCR score of 2.02 (SD = 7.43) indicated these articles were cited more than twice as often as comparable NIH-funded papers. Regarding translational content, articles had a mean APT score of 0.52 (SD = 0.31), indicating that overall, the likelihood an article will be translated to clinical research via citation in a clinical article is 52%. Thus far, articles in the publication portfolio have been cited an average of 33.7 times each, totaling 1,017,291 times cited, with 13,012 articles (56.9%) being cited by clinical articles. Regarding translational stages, represented in the Triangle of Biomedicine, the articles’ contents averaged 79% human-oriented, 11.7% molecular/cellular-oriented, and 7.3% animal-oriented content. A total of 4,809 (15.9%) were referenced in Overton-indexed policy literature by January 2024. Many were referenced more than once, totaling 13,191 references. As can be seen in Table 1, there were statistically significant differences between articles supported by KL2, TL1, and both KL2 and TL1 grants. In general, K-supported publications had higher metric scores than T-supported publications, but articles that reported funding from both KL2 and TL1 grants often had similar or higher metrics than by themselves.

**Table 1.** Bibliometrics for CTSA-supported KL2 and TL1 publications

Bibliometric	KL2-supported publication (n = 20,546), mean (SD)	TL1-supported publication (n = 7,995), mean (SD)	KL2 & TL1-supported publication (n = 1,676), mean (SD)	Statistical Significance
Publication year	2016.72 (4.04)	2018.23 (3.55)	2015.54	*p* < 0.001
Journal impact factor (JIF)	5.85 (8.61)	5.72 (6.77)	5.21 (9.06)	*p* = 0.014
Approximate potential to translate (APT)	0.55 (.31)	0.45 (.31)	0.54 (.31)	*p* < 0.001
% Clinical Articles	9.96%	5.84%	12.65%	*p* < 0.001
% Cited by Clinical Article	46.94%	31.46%	50.89%	*p* < 0.001
% Human Research	0.83 (.32)	0.70 (.40)	0.80 (.34)	*p* < 0.001
% Animal Research	0.05 (.18)	0.12 (.26)	0.06 (.19)	*p* < 0.001
% Molecular/Cellular Research	0.10 (.21)	0.16 (.27)	0.11 (.22)	*p* < 0.001
Relative citation ratio (RCR)	2.02 (4.97)	1.89 (3.70)	2.50 (24.82)	*p* = 0.011
Number of academic citations	34.62 (99.14)	26.71 (67.27)	55.19 (687.59)	*p* < 0.001
Cited by policy n (%) Overton	3634 (17.7%)	862 (10.8%)	313 (18.7%)	*p* < 0.001

Abbreviation: CTSA, Clinical and Translational Science Award.

To date, the mean AAS score for the publication portfolio is 28.9 (SD = 191.04). Although 3,083 articles have received no altmetric attention, a sizable group of articles (4,625, 18.5%) have received scores of 20 or higher and a select group of 63 articles had AAS scores of 1,000 or more (AAS range: 1,004-19,660). Specific altmetrics included early mentions in public/community sources: over 64K news articles, 7K blog posts, and 480K X posts; and early attention in academic sources: and over 1.8 million downloads by Mendeley readers. Meanwhile, longer-term altmetric attention included 3,357 policy document references, 3,188 Wikipedia page references, and 6,384 references in patent applications. Table 2 includes altmetric descriptive statistics for the publication portfolio and comparisons between grant mechanisms. There are statistically significant differences for several metrics, but the wide standard deviations for many of the metrics indicate substantial skew in the altmetric attention.

**Table 2.** Short- and long-term academic and altmetric impact measures for Clinical and Translational Science Awards-supported KL2 and TL1 publications

Bibliometric	KL2-supported publication (n = 20,546), mean (SD)	TL1-supported publication (n = 7,995), mean (SD)	KL2 & TL1-supported publication (n = 1,676), mean (SD)	Significance
Altmetric attention score (AAS)	29.10 (131.72)	30.40 (305.46)	19.63 (71.75)	*p* = 0.164
Short-term impact				
News stories	2.71 (14.74)	2.39 (20.07)	1.71 (9.90)	*p* = 0.050
Blog posts	0.30 (1.25)	0.25 (1.38)	0.33 (1.34)	*p* = 0.014
X posts	17.25 (126.46)	26.45 (485.74)	11.19 (40.36)	*p* = 0.033
Mendeley downloads	75.78 (140.64)	66.70 (118.98)	84.09 (279.69)	*p* < 0.001
Long-term impact				
Policy documents	0.15 (.77)	0.09 (.58)	0.18 (.77)	*p* < 0.001
Patent applications	0.25 (2.65)	0.26 (2.08)	0.35 (2.34)	*p* = 0.36
F1000 Peer review mentions	0.01 (.21)	0.01 (.21)	0.02 (.40)	*p* = 0.64
Wikipedia articles	0.15 (5.53)	0.07 (.47)	0.13 (.99)	*p* = 0.53

### Part 2: Case examples of CTSA-supported research that generated high levels of interest and impact outside academic spheres (“Influencers”)

For a selection of articles, we investigated characteristics of CTSA-supported research that garnered high levels of public attention. These “Influencer” articles were selected as a cross-section from the group of 63 articles that attained AAS scores higher than 1,000, which were higher scores than 99.7% of all articles. Illustrative examples were chosen to show variability in research category, supporting CTSA hub, grant mechanism, content, and time period. Articles fell into seven general categories including: COVID-19, Diet & Exercise, Drug overdose, Genetics, Alzheimers/Mental Health/Cognition, Risk or disease burden, Public health. The greatest proportion (18/63, 28.6%) were published after 2020 and were directly related to the COVID-19 epidemic, indicating high public attention on this important health topic. Table 3 shows the impact of the CTSA by reporting details of these research articles based on CTSA-supported research, including their author and bibliometric information, the category of research, a short summary of the article, and the number and type of altmetrics that were impacted by the article. Interestingly, articles generated differing levels of interest across various altmetrics and traditional academic bibliometric indicators. Summaries of papers with AAS > 1000 by research area are available as Supplemental Digital Appendix 1.

**Table 3.** Summaries of representative papers with Altmetric Attention Score (AAS) scores>1000

Article	CTSA Support	Research Area	Bibliometrics	Summary
An evidence review of face masks against COVID-19. Proc Natl Acad Sci U S A. 2021 Jan 26;118(4):e2014564118. [61]	TL1 TR001880 Institute for Translational Medicine and Therapeutics, University of Pennsylvania	Infectious Diseases (COVID-19)	AAS: 19,660 (news: 742, X: 35,728, blogs: 42, Mendeley: 1,498) CNCI: 39.49 JIF: 12.78 Overton Citations: 36 Academic Citations: 567	This study is a narrative review framework for examining mask usage, particularly, to inform the following: the impact of mask usage, characteristics of transmission of COVID-19, how masks protect the wearer, how masks reduce the spread of COVID-19 if worn by infected persons, and sociological and implementation considerations of mask usage. This article concludes with strong recommendations for public officials and governments to encourage the widespread use of face masks in public.
Global Burden of Disease Study 2013 Collaborators. Global, regional, and national incidence, prevalence, and years lived with disability for 301 acute and chronic diseases and injuries in 188 countries, 1990-2013: a systematic analysis for the Global Burden of Disease Study 2013. Lancet. 2015 Aug 22;386(9995):743-800. [51]	KL2 TR001088 Dartmouth Synergy Clinical and Science Institute	Psychiatry (Risk of Acute & Chronic Disease Burden)	AAS: 2,500 (news: 291, X: 1,271, blogs: 25, Mendeley: 6,193) CNCI: 133.01 JIF: 44.00 Overton Citations: 14 Academic Citations: 4,234	This study utilized 35,620 data sources to estimate the burden of 301 diseases and injuries, along with 2,337 sequelae. The authors conducted a comorbidity simulation to estimate the number of concurrent sequelae experienced by individuals, by country, year, age, and sex. Findings highlight that the global aging population is significantly increasing the number of individuals living with the aftereffects of diseases and injuries. Although mortality rates are declining, the rates of years lived with disabilities are decreasing at a much slower pace. This suggests that health systems will need to focus increasingly on managing the non-fatal aspects of diseases and injuries. The shift towards non-fatal outcomes as the dominant source of burden of disease is happening rapidly across most regions, except in sub-Saharan Africa.
Rapid Sequencing-Based Diagnosis of Thiamine Metabolism Dysfunction Syndrome. N Engl J Med. 2021 Jun 3;384(22):2159-2161. [62]	TL1 TR001113 Scripps Research Translational Institute	Genetics & Heredity (Neurology/ Pediatrics)	AAS: 2,139 (news: 14, X: 5,705, blogs: 3, Mendeley: 53) CNCI: 7.08 JIF: 176.08 Overton Citations: 2 Academic Citations: 38	This article shows the fulfillment of the promise of the Human Genome Project to transform health care. The authors sequenced the genome of an infant with encephalopathy in just over 11 hours. The results led to a clinical diagnosis of thiamine metabolism dysfunction syndrome 2 (THMD2) 16.5 hours after a blood sample was obtained and 13 hours after sequencing was initiated. Fortunately, the diagnosis was treatable. This case shows the potential for decreased suffering and improved outcomes by using rapid genome sequencing in a multidisciplinary, integrated, precision medicine delivery system. Such a system includes identification of infants with suspected genetic diseases on the day of admission, rapid genome sequencing as a first-tier test, communication of results in a way that guides fast treatment based on the genetic sequencing results.
Changing dynamics of the drug overdose epidemic in the United States from 1979 through 2016. Science. 2018 Sep 21;361(6408):eaau1184. [63]	KL2 TR001856 University of Pittsburgh Clinical and Translational Science Institute	Public, Environmental & Occupational Health (Drug Overdose)	AAS: 1,602 (news: 154, X: 1,773, blogs: 17, Mendeley: 331) CNCI: 24.85 JIF: 41.06 Overton Citations: 56 Academic Citations: 341	This article reports the analysis of records of 599,255 deaths from 1979 through 2016 from the National Vital Statistics System in which accidental drug poisoning was identified as the main cause of death. Results of the analysis highlight a pattern which shows predictable growth over the last 38 years, suggesting that the epidemic will continue this way for several more years. The growth was shown to be a result of a composite of multiple distinctive sub-epidemics of different drugs (primarily prescription opioids, heroin, methadone, synthetic opioids, cocaine, and methamphetamine), each with its own demographic and geographic characteristics. The results are reported to illustrate the forces holding the sub-epidemics together. This understanding is important to prevention and intervention strategies.
Global, regional, and national age-sex specific all-cause and cause-specific mortality for 240 causes of death, 1990-2013: a systematic analysis for the Global Burden of Disease Study 2013. Lancet. 2015 Jan 10;385(9963):117-71. [64]	KL2 TR001088 Dartmouth Synergy Clinical and Science Institute	Public, Environmental & Occupational Health (Mortality)	AAS: 1,428 (news: 63, X: 1,140, blogs: 16, Mendeley: 3,306) CNCI: 224.86 JIF: 44.00 Overton Citations: 706 Academic Citations: 4,952	The study focused on evaluating the evidence concerning age and sex-specific trends in all-cause and cause-specific mortality rates. The authors employed the Global Burden of Disease 2010 methods, with some improvements for enhanced accuracy, using updated data from vital registrations, surveys, and censuses. The general trend observed in most countries indicates a reduction in age-sex specific mortality, accompanied by a shift towards a higher proportion of deaths caused by non-communicable diseases and injuries. However, the study notes that whether convergence across countries is apparent depends on whether absolute or relative measures of inequality are considered. Additionally, the study identified an increase in age-standardized death rates for seven major causes.
Transcription factors operate across disease loci, with EBNA2 implicated in autoimmunity. Nat Genet. 2018 May;50(5):699-707. [65]	KL2 TR001426 Cincinnati Center for Clinical and Translational Science and Training	Genetics & Heredity (Chronic Disease)	AAS: 1,037 (news: 114, X: 470, blogs: 12, Mendeley: 447) CNCI: 9.56 JIF: 25.46 Overton Citations: 0 Academic Citations: 204	This is a genetic study of transcription factors, or proteins that help turn specific genes “on” or “off” by binding to nearby DNA. Transcription factors that are activators turn genes on. Repressors lower or turn genes off. Many of these transcription factors were found at many locations and were associated with complex disorders like prostate and breast cancer. Strikingly, this paper shows gene-environment interaction in that proteins from Epstein-Barr virus, which cause mononucleosis, were associated with multiple autoimmune gene locations in lupus erythematosus, multiple sclerosis, rheumatoid arthritis, inflammatory bowel disease, type 1 diabetes, juvenile idiopathic arthritis and celiac disease. This implies genetic mechanisms dependent on Epstein-Barr virus suggesting new models for disease origins.
Efficacy of commercial weight-loss programs: an updated systematic review. Ann Intern Med. 2015 Apr 7;162(7):501-12. [66]	TL1 TR001078 KL2 TR001077 Johns Hopkins Institute for Clinical and Translational Research	Nutrition (Diet/Weight Loss)	AAS: 1,024 (news: 177, X: 180, blogs: 18, Mendeley: 357) CNCI: 3.12 JIF: 16.59 Overton Citations: 8 Academic Citations: 211	This is a meta-analysis of 45 different studies, 39 of which were RCTs. The study compared Weight Watchers, Jenny Craig, Nutrisystem, Very-low-calorie programs, SlimFast and the Atkins diet. Most studies were short (<12 months) had a high dropout rate and lacked blinding. The overall results showed that Jenny Craig and Weight Watchers appeared to show the most weight loss (4.9% and 2.6%, respectively) over 12 months compared to control/education and counseling. Nutrisystem showed promise (3.8% more weight loss) as did very-low-calorie programs (4.0%), but both were shorter studies and sustained loss was not shown. Atkins results were the lowest loss (0.1%-2.9% loss) over 12 months and results for SlimFast were mixed. The paper concluded that Jenny Craig and Weight Watchers were the programs that were worth referring patients to based upon the longer studies’ results.

As an illustrative example, the Institute for Translational Medicine and Therapeutics at the University of Pennsylvania, through their TL1 program, partially supported research reported in an article which reviewed mask usage to inform characteristics of COVID-19 and how masks protect the wearer and reduce the spread of COVID-19 [36], published in the *Proceedings of the National Academy of Sciences* (*PNAS*) in 2021. As of January 2024, this article had amassed the largest number of altmetric references in the CTSA training portfolio, with an AAS score of 19,660 being tweeted over 35,000 times, posted on 42 blogs, and being included in 742 news articles, including stories published by the Atlantic, Scientific American, The Washington Post, Time magazine, and a variety of online news outlets. One example reference appeared in the New York Times and was titled *One Mask is Good. Would Two be Better?* [50], an article reporting on the evidence for wearing face masks to slow the spread of COVID-19, the type of masks that that are recommended, and the potential benefits of wearing more than one mask. The article used the publication as evidence that research across several scientific fields supported the widespread use of masks to halt the transmission of COVID-19.

As a second illustrative example, the Dartmouth SYNERGY Clinical and Translational Science Institute, through their KL2 program, partially supported research which estimated the global burden of 301 diseases and injuries [51], published in the Lancet in 2105. As of January 2024, this article had an AAS score of 2,500 being tweeted over 1,200 times, posted on 25 blogs, and being included in 291 news articles, including stories published by the New York Times, National Public Radio, BBC news, and Time magazine. One of these articles, published in the New York Times was titled *Lives Grow Longer, and Health Care’s Challenges Change* [52], reported the major findings from the study and interpreted related implications for public health in various global settings.

### Part 3: Bibliometric characteristics that influence public attention

Table 4 shows results from the analysis comparing classifications of altmetric attention (no attention, moderate attention, and high attention). Results indicate that those that had a higher JIF (*OR* = 1.12, 95% *CI* 1.11 – 1.12; *p* < .001), were published after 2020 (*OR* = 1.56, 95% *CI* 1.43 – 1.718; *p* < .001), received more Mendeley downloads (*OR* = 1.01, 95% *CI* 1.006 – 1.007; *p* < .001), had higher RCR scores (*OR* = 1.46, 95% *CI* 1.41 – 1.51; *p* < .001), have been cited by a policy document (*OR* = 1.45, 95% *CI* 1.34 – 1.58; *p* < .001), and had accrued less academic citations (*OR = 0.986*, 95% *CI* 0.985 – 0.988; *p* < .001), were more likely to receive higher levels of altmetric attention. Conversely, articles with lower APT scores were less likely to receive altmetric attention (*OR* = 0.72, 95% *CI* 0.67 – 0.77; *p* < .001).

**Table 4.** PLUM regression predicting 3 levels of altmetric attention

		OR	OR lower	OR upper	Significance
Threshold	AAS = 0.00	0.39	0.33	0.45	<0.001
	AAS = 1.00	22.71	19.53	26.39	<0.001
Location	Journal Impact Factor (JIF)	1.12	1.11	1.12	<0.001
	Year Post 2020	1.56	1.43	1.71	<0.001
	Year (Pre 2020 referent)	1.00	1.00	1.00	
	Mendeley Downloads	1.01	1.006	1.007	<0.001
	Relative Citation Ratio (RCR)	1.46	1.41	1.51	<0.001
	Total Citations	0.986	0.985	0.988	<0.001
	Cited KL2 only	0.097	0.85	1.11	0.637
	Cited TL1 only	1.09	0.95	1.26	0.210
	Cited KL2 and TL1 (referent)	1.00	1.00	1.00	
	Cited by Policy (Yes)	1.45	1.34	1.58	<0.001
	Cited by Policy (No - referent)	1.00	1.00	1.00	
	Approximate potential to translate (APT)	0.72	0.67	0.77	<0.001
	Approximate potential to translate (APT) (High - referent)	1.00	1.00	1.00	

## Discussions

Zerhouni et al, in the pioneering articles outlining the CTSA program, laid out an ambitious plan for evaluation and gauging of impact which can be applied both within and beyond CTSA hubs [53–55]. Key to this plan was the recognition that science occurs in stages and has impact that unfolds over time. Additional vital elements were a focus on training scholars and trainees to become investigators, working across the borders of CTS, and understanding processes and science itself with its bidirectional flow of information and advancement. What was especially visionary was the idea that translational science would become an “integral and essential part of health-care delivery” and that CTS had the potential to increase “public awareness and trust in clinical research [53]. One way that this call to action has been met is through the consistent application of the 3 Ds framework- Developing, Demonstrating, and Disseminating [16,56] translational science advances through scientific publications.

Past research has used bibliometric methods to examine aspects of dissemination and impact of the CTSA program [1,2,4,9–11]. However, this is the first study that focuses at this scale on CTSA scholars and trainees and links efforts to support translational science to subsequent altmetric impact, verifying the dissemination of research to the public and across academia. This method represents a valuable approach to evaluating training outcomes and provides important considerations for the establishment of communication and dissemination training and dissemination programs through academic medical centers. By demonstrating how supported research has influence beyond academia, this study represents a significant advance in our ability to evaluate translational research impact. However, we caution that each bibliometric and altmetric indicator has their strengths and weaknesses so, consistent with previous studies, we recommend integrating a complementary range of metrics and approaches to provide a full picture of the impact of research [9,11]. We used three complementary approaches to examine Academic Influencers and the associations between traditional bibliometrics and altmetrics connected with the CTSA training grant supported publication portfolio. Results revealed many altmetric references to CTSA- supported research and contributions to public discourse on COVID-19. We connected direct evidence of CTSA support to public health outcomes of national and international interest. Our results confirm that although many scientific publications receive no or little attention, many publications generate attention both inside and outside academia. Findings indicate differing levels of bibliometric and altmetric indicators related to the grant mechanism which was cited. However, no consistent pattern or hierarchy of metric scores was found among grants mechanisms. Future research should systematically investigate these differences to determine the sources of this variability. We also found that the kinds of publications that influence this attention were more likely to: (1) be published after 2020, (2) be cited by a policy document, (3) receive slightly less academic citations (a finding likely related to the time needed to accrue academic citations), (4) be more human-centered research and (5) show greater academic influence through metrics including citations ratios relative to similar articles, Mendeley downloads, and publishing in higher impact journals.

One major factor that influenced altmetric attention was being published after the year 2020. Although one could argue that altmetric attention will increase as society becomes more interested in science and is more connected through social media, we attribute much of this finding to the COVID-19 epidemic and public recognition that research was critical to the immediate public health needs of navigating and fighting the pandemic. Other COVID-19 bibliometric research has found high levels of publication and citation activity without a corresponding increase in retractions, high levels of altmetric attention, greater research uptake into policy, and accelerated translation related to the pandemic [1,57–60]. In essence, COVID-19 research was high-quality overall and used by other researchers, the public and policymakers at an accelerated pace. We will also note that this study builds on previous research which has found that the CTSA publication portfolio covers a diversity of academic journals and research fields [2,11]. The research of translational scientists is clearly being translated to other research areas and disciplines. Consistent with previous recommendations [2], we suggest that future research examine overlap and intersections of research areas to give a comprehensive view of impact beyond translational science. Future research should closely track and interpret public health interest in science by examining increases in the altmetric attention among newly popular health topics (e.g. new weight loss drugs).

Advancing science through publications and their corresponding impacts on health and society is a living and iterative process. Understanding advances represented in a publication portfolio has advantages for scholars and trainees, institutions, and the granting agencies which support them. First, we hope that each of these groups will have a greater awareness that publications are being viewed and discussed by those outside the traditional academic community. Second, researchers should prepare to explain their research findings to diverse audiences who may need tailored messaging to understand the implications of completed research. Third, it may be advantageous for researchers to write in a way that is accessible to the public or include a section of summarized results that represent the overall findings from research studies. At the institutional or granting agency level, programs which summarize and communicate research findings should be developed, implemented and evaluated to ensure sufficient dissemination. It is also advantageous for science to document its development and for the public to recognize the state of scientific progress. Results from this study demonstrate nearly 20 years of scientific influence across society through production, growth, and impact as demonstrated by traditional and altmetric measures. Although there are challenges to evaluate the dissemination of research into society, emerging methods have made it possible to connect academic literature to public influence. It is likely that the further development of artificial intelligence will be an invaluable tool to analyze and report on this impact.

Limitations of this study include those common to tracking publications attributed to grant-supported research. For instance, it is likely that not all investigators acknowledge their grant support and not all journals are indexed in PubMed, which aims to index all NIH-funded research. If authors cite their grants when publishing an article, then the article is expected to be indexed in PubMed; however, the requirement to cite funding sources is difficult to universally enforce and there is a possibility of errors and omissions. Second, not all publications are represented across bibliometric indices, resulting in missing data as some metrics need time to be generated and gain stability (i.e., RCR is only available for articles older than 1 year; JIF is limited to journals meeting Web of Science journal evaluation criteria). Therefore, it is important to assess publication portfolios through several metrics and approaches which present converging evidence, such as in this study. Third, bibliometrics are indices of the subsequent use and popularity of publications, not of the quality of the science itself. Large scale studies, such as this analysis, are unable to discern the quality of the science represented in individual articles. A limitation specific to altmetrics is that current metrics do not capture all public attention paid to research articles. Additionally, although many altmetrics are available sooner than traditional citation metrics, some, such as patent and policy references, may accrue some time after publication. Further, the content and quality of altmetric attention can vary, or may not have strong relevance for translational advancement. Lastly, the publications drawn for this study are linked to training grant support, indicating authorship by trainees. It is likely that they had varying degrees of leadership on the articles.

## Conclusion

This paper provides an expansive view of an NIH-supported training grant bibliometric portfolio and is complemented with case studies that exhibit the highest altmetric impact articles. Future research should use similar methods to examine how cross-institute NIH-support mechanisms accelerate translation via specific health content areas to comprehensively understand research and clinical advancements. For instance, this could include an investigation of support for research areas such as substance use and abuse or health equity and access to understand how large-scale federal support is applied and results in publications and public impact. Concurrently, these studies have the potential to investigate the predictive accuracy of grant proposal peer-review scores, size and type of award (e.g. U-award, R01, R21, vs. pilot grants), and co-sponsorship in relation to return on investment in terms of science advancement and dissemination. Beyond research, CTS leaders and investigators should realize that crucial aspects of translating science are engaging the public, educating the public, and undertaking research which is increasingly relevant to localized health priorities and needs. Therefore, as an initial step, it is incumbent upon scientists, institutions, and granting agencies to emphasize science communication training programs for scholars and trainees that include implementing dissemination strategies that clearly, promptly, and accurately convey research findings and track their influence within public discourse.

## Supporting information

10.1017/cts.2025.10067.sm001 Nehl et al. supplementary material Nehl et al. supplementary material

## Supplementary material

The supplementary material for this article can be found at https://doi.org/10.1017/cts.2025.10067
