# Supplementary material for: Academic influencers: Clinical and Translational Science scholars and trainees at the intersection of influential scholarship and public attention
Source: J Clin Transl Sci. 2025 Jun 2;9(1):e130. doi: 10.1017/cts.2025.10067 (PMC12260972; doi:10.1017/cts.2025.10067)
Supplement: Nehl et al. supplementary material [file S2059866125100678sup001.docx]

| **Supplemental Table 1:**  **Summaries of papers with AAS > 1000 by research area** | |
| --- | --- |
| **Research Area** | **Reference** |
| A. INFECTIOUS DISEASES  (COVID-19) | 1. The airborne lifetime of small speech droplets and their potential importance in SARS-CoV-2 transmission. NAS 2020. 2. Visualizing speech-generated oral fluid droplets with laser light scattering. NEJM 2020. 3. Outpatient treatment of COVID-19 and incidence of post-COVID-19 condition over 10 months (COVID-OUT): A multicentre, randomised, quadruple-blind, parallel-group, Phase 3 trial. Lancet Infectious Disease 2023. 4. Randomized trial of metformin, ivermectin, and fluvoxamine for COVID-19. NEJM 2022 5. SARS-CoV-2 infection of the oral cavity and saliva. Nature Medicine 2021 6. Multisystem inflammatory syndrome in U.S. children and adolescents. NEJM 2020 7. Saliva or nasopharyngeal swab specimens for detection of SARS-CoV-2. NEJM 2020 8. Microvascular injury in the brains of patients with COVID-19. NEJM 2021 9. Diverse functional autoantibodies in patients with COVID-19. Nature 2021 10. Spike mutation D614G alters SARS-CoV-2 fitness. Nature 2021 11. We shouldn't worry when a virus mutates during disease outbreaks. Nature Microbiology 2020 12. Saliva viral load is a dynamic unifying correlate of COVID-19 severity and mortality. Preprint 2021 13. Functional SARS-CoV-2 specific immune memory persists after mild COVID-19. Cell 2021 14. Growing public health concern of COVID-19 chronic olfactory dysfunction. JAMA Otolaryngology Head Neck Surg. 2022 15. COVID-19 and dementia: Analyses of risk, disparity, and outcomes from electronic health records in the US. Alzheimers Dement. 2021 16. Metformin reduces SARS-CoV-2 in a Phase 3 randomized placebo controlled clinical trial. Preprint 2023 17. Performance of rapid antigen tests to detect symptomatic and asymptomatic SARS-CoV-2 infection. Annals of Internal Medicine 2023 18. Long-term health consequences of COVID-19. JAMA 2020 |
| B. PSYCHIATRY  (Risk of Acute & Chronic Disease Burden) | 1. Global, regional, and national comparative risk assessment of 79 behavioural, environmental and occupational, and metabolic risks or clusters of risks in 188 countries, 1990-2013: A systematic analysis for the Global Burden of Disease Study 2013. Lancet 2015 2. Olfactory dysfunction predicts 5-year mortality in older adults. PlosOne 2014 3. Subconcussive head impact exposure and white matter tract changes over a single season of youth football. Radiology 2016 4. Beyond "median waiting time": Development and validation of a competing risk model to predict outcomes on the kidney transplant waiting list. Transplantation 2016 |
| C.GENETICS & HEREDITY (Neurology/ Pediatrics) | 1. Rare and low-frequency coding variants alter human adult height (Nature 2017) 2. A saturated map of common genetic variants associated with human height (Nature 2022) 3. Evidence for a role of the oxytocin system, indexed by genetic variation in CD38, in the social bonding effects of expressed gratitude ( Soc Cogn Affect Neurosci. 2014) 4. The trans-ancestral genomic architecture of glycemic traits. (Nat. Genetics 2021) |
| D.PUBLIC, ENVIRONMENTAL & OCCUPATIONAL HEALTH  (Drug Overdose) | 1. National Trends in Hospitalizations for Opioid Poisonings Among Children and Adolescents, 1997 to 2012. JAMA Pediatrics. 2016 2. A non-hallucinogenic psychedelic analogue with therapeutic potential. Nature, 2021. |
| E.PUBLIC, ENVIRONMENTAL & OCCUPATIONAL HEALTH (Mortality) | 1. Genome-wide polygenic scores for common diseases identify individuals with risk equivalent to monogenic mutations. Nat Genet. 2018. 2. Olfactory dysfunction predicts 5-year mortality in older adults. PloS One 2014. 3. Subconcussive Head Impact Exposure and White Matter Tract Changes over a Single Season of Youth Football. Radiology, 2016. 4. Beyond "Median Waiting Time": Development and Validation of a Competing Risk Model to Predict Outcomes on the Kidney Transplant Waiting List.. Transplantation 2016. |
| F.GENETICS & HEREDITY  (Chronic Disease) | 1. A saturated map of common genetic variants associated with human height.Nature, 2022. 2. Evidence for a role of the oxytocin system, indexed by genetic variation in CD38, in the social bonding effects of expressed gratitude. Soc Cogn Affect Neurosci., 2014. 3. The trans-ancestral genomic architecture of glycemic traits. Nat Genet. 2021. 4. Rare and low-frequency coding variants alter human adult height. Nature 2017. |
| G.NUTRITION  (Diet/Weight Loss) | 1. The effects of sleep extension on the athletic performance of collegiate basketball players. Sleep, 2011. 2. Changes in Body Mass Index Among School-Aged Youths Following Implementation of the Healthy, Hunger-Free Kids Act of 2010. JAMA Pediatrics, 2023. 3. Chocolate intake and risk of clinically apparent atrial fibrillation: the Danish Diet, Cancer, and Health Study. Heart, 2017. 4. Early Time-Restricted Feeding Improves Insulin Sensitivity, Blood Pressure, and Oxidative Stress Even without Weight Loss in Men with Prediabetes. Cell Matab. 2018. |
